# Myocardial infarction size as an independent predictor of intramyocardial haemorrhage in acute reperfused myocardial ischaemic rats

**DOI:** 10.1186/s40001-022-00834-5

**Published:** 2022-10-28

**Authors:** Rui Xia, Tong Zhu, Yu Zhang, Bo He, Yushu Chen, Lei Wang, Yang Zhou, Jichun Liao, Jie Zheng, Yongmei Li, Fajin Lv, Fabao Gao

**Affiliations:** 1 Department of Radiology, The First Affiliated Hospital of Chongqing Medical University, 1 Youyi Road, Chongqing, 400042 China; 2 Department of Radiology, TongJi Hospital, TongJi Medical College, HuaZhong University of Science & Technology, Wuhan, 430030 China; 3 Department of Radiology, West China Hospital, Sichuan University, Chengdu, 610041 China; 4 Department of Pathology, Chongqing Medical University, Chongqing, 400016 China; 5 Mallinckrodt Institute of Radiology, Washington University School of Medicine in St. Louis, St. Louis, USA

**Keywords:** Intramyocardial haemorrhage, Myocardial infarction, Magnetic resonance imaging

## Abstract

**Background:**

In previous studies, haemorrhage occurred only with large infarct sizes, and studies found a moderate correlation between the extent of necrosis and haemorrhage, but the extent of infarction size in these studies was limited. This study aimed to find the correlations between intramyocardial haemorrhage (IMH), myocardial infarction (MI), and myocardial oedema (ME) from small to large sizes of MI in a 7.0-T MR scanner.

**Methods:**

Different sizes of myocardial infarction were induced by occluding different sections of the proximal left anterior descending coronary artery (1–3 mm under the left auricle). T2*-mapping, T2-mapping and late gadolinium enhancement (LGE) sequences were performed on a 7.0 T MR system at Days 2 and 7. T2*- and T2-maps were calculated using custom-made software. All areas were expressed as a percentage of the entire myocardial tissue of the left ventricle. The rats were divided into two groups based on the T2* results and pathological findings; MI with IMH was referred to as the + IMH group, while MI without IMH was referred to as the –IMH group.

**Results:**

The final experimental sample consisted of 25 rats in the + IMH group and 10 rats in the –IMH group. For the + IMH group on Day 2, there was a significant positive correlation between IMH size and MI size (r = 0.677, P < 0.01) and a positive correlation between IMH size and ME size (r = 0.552, P < 0.01). On Day 7, there was a significant positive correlation between IMH size and MI size (r = 0.711, P < 0.01), while no correlation was found between IMH size and ME size (r = 0.429, P = 0.097). The MI sizes of the + IMH group were larger than those of the –IMH group (P < 0.01).

**Conclusions:**

Infarction size prior to reperfusion is a critical factor in determining IMH size in rats.

## Background

Emergency percutaneous coronary intervention in acute myocardial infarction (MI) restores epicardial coronary blood flow, but intramyocardial haemorrhage (IMH) is still observed in up to 50% of patients [1]. The cardiovascular magnetic resonance imaging (CMR) T2* sequence allows for more accurate visualization of the IMH region than the T2 sequence [2–5].

However, IMH and microvascular obstruction (MVO) showed close anatomic correlation [6]. Recently, researchers have shown that IMH is more closely associated with adverse outcomes than MVO [7], which is an irreversible pathological consequence of severe reperfused myocardial injury [8]. In previous studies, haemorrhage occurred only with the largest infarctions [2, 7, 9–22]. A few studies found a moderate correlation between the extent of necrosis and haemorrhage [2, 19–21], but the extent of infarction size in these studies was limited. We hypothesized that IMH could occur in small infarction sizes and that there was a close correlation between IMH size and MI size in small and larger myocardial infarction sizes, which can be assessed using CMR images.

In this study, we intended to induce different sizes of myocardial infarction created by occluding different sections of the proximal left anterior descending coronary artery in a rat model. CMR imaging, including T2, T2*, and LGE, was performed on these rats on Days 2 and 7 after reperfused myocardial ischaemia. The study had four main aims: (1) to determine whether IMH is present in rats with small MI sizes; (2) to determine the relationship between IMH, MI, and myocardial oedema (ME) sizes; (3) to compare T2 and T2* values for IMH, MI, ME, and remote areas; and (4) to measure changes in T2 and T2* values and sizes of IMH, MI, and ME from Day 2 to Day 7.

## Methods

### Animal models

This study was approved by the Institutional Animal Care and Use Committee of West China Hospital of Sichuan University. Forty-five female Sprague–Dawley rats with body weights between 250 and 350 g were investigated. Prior to surgery, the rats were anaesthetized intraperitoneally with sodium pentobarbital (50 mg/kg), and respiration was maintained with a rodent ventilator. A real-time electrocardiogram was monitored throughout surgery.

To introduce the coronary occlusion, a thoracotomy was performed. The chest was opened at the fourth intercostal space to expose the heart. The pericardium was opened with forceps, and a 6.0 suture was passed underneath the left anterior descending coronary artery (LAD) at 1–3 mm [22, 23]. This resulted in the occlusion of the different sections (n = 15 for 1–1.5 mm, 2 mm, and 2.5–3 mm) of the proximal left anterior descending coronary artery, leading to different sizes of myocardial infarction for each rat. Coronary occlusion was achieved by tightening the suture over a 3.0 suture. All occlusions were maintained for 60 min, and reperfusion was achieved by untying the knot and releasing the suture from the occlusion [24, 25]. Reperfusion was confirmed by ECG changes. The duration time of reperfusion was calculated from the release of the ligature, while Day 2 and Day 7 were selected as the acute and subacute phases, respectively.

## MRI protocols

All MRI protocols were implemented on a 7.0-T MR system (BRUKER BIOSPEC 70/30). More than five (depending on heart size) single-slice MSME (multislice multiecho)-T2-mapping and MGE (multigradient-echo)-T2*-mapping images with the same slices were acquired on the short-axis slices during the mid-diastolic phase and end-inspiratory period using both ECG and respiratory gating systems (SA Instruments, Inc.). Late gadolinium enhancement (LGE) imaging was performed by fast imaging with steady-state precession (FISP)-cine on the same slice locations at 10 min after an injection of Gd-DTPA (Magnevist, Bayer Health Care Pharmaceuticals, 0.15 mmol/kg).

The imaging parameters included T2-mapping: TR/TE = 1500 ms/10, 20, 30 ms, matrix size = 192 × 192, FOV = 50 × 50 mm, and slice thickness = 1.5 mm without a slice gap; T2*-mapping: FA (Flip angle) = 30°, TR/TE = 1000 ms/3.5,7,10.5,14,17.5,21,24.5,28 ms, matrix size = 192 × 192, FOV = 50 × 50 mm, and slice thickness = 1.5 mm without slice gap; and LGE: TR/TE = 5.2 ms/1.8 ms, FA = 25°, matrix size = 256 × 256, FOV = 50 × 50 mm, slice thickness = 1.5 mm, 25 frames for each slice.

## Histology

After the MRI scans, the rats were killed with potassium chloride, and the hearts were rapidly excised. Each heart was cut into five or more transverse slices from apex to base. Each slice was approximately 1.5 mm thick to match MRI slices. These slices were then incubated in 4% paraformaldehyde for haematoxylin and eosin staining.

## Data analysis

According to the T2* results and pathological findings, rats with both MI and IMH were assigned to a group referred to as the + IMH group, while rats with MI but no IMH were assigned to a group referred to as the –IMH group.

T2*- and T2-maps were calculated using custom-made software written in MATLAB 7.1 (The Mathworks). ME was defined as high T2 values (> mean ± 2 SD in remote normal tissue areas) in T2 maps, and MI areas were identified as positive enhanced areas (> mean ± 5 SD in remote normal tissue areas) in LGE images. The areas of haemorrhage were identified as a hypointense core (at least 2 standard deviations less than the perihaemorrhagic myocardium) within a hyperintense territory (confirmed by an LGE-positive area) on T2* maps. ME, MI, and IMH sizes were added slice-by-slice and expressed as a percentage of the whole myocardial tissue of the left ventricle (%LV) [26, 27].

More than one slice was selected to calculate the mean T2 and T2* values for each rat. The absolute differences in T2* and T2 were computed as the respective differences in the values between myocardial injury regions of interest (ME, MI, and IMH) and remote myocardium; they were labelled T2* and T2. The corresponding relative differences were computed by normalizing the absolute differences using the values from remote regions and multiplying by 100%; they were then labelled Relative T2* (%) and Relative T2 (%).

All statistical analyses were performed using SPSS Statistics, version 23.0 (IBM Corp.). Quantitative data were tested for normal distribution using the Kolmogorov–Smirnov test. Data are expressed as the mean ± SD or as the median (25%–75% interquartile range). P values < 0.05 were considered significant. The correlations between IMH and the size of MI or ME were assessed using Spearman analysis in the + IMH group. Datasets following normal distributions (T2 and T2* values) were compared using analysis of variance with Student–Newman–Keuls post hoc analysis. When comparing the T2 and T2* values for Days 2 and 7, paired-samples t tests were performed. Comparisons between values of size were performed using nonparametric tests.

## Results

### Animals

Thirty-five animals were available for imaging (three died during reperfusion, two died during MR scanning, four had no MI, and one had uninterpretable image quality). There were significant differences in ME, MI, and IMH upon ligating different sections of the proximal left anterior descending coronary artery (Table 1).

**Table 1:** The size parameters of the different ligating left anterior descending coronary artery groups

	1–1.5 mm (%) n = 11	2 mm (%) n = 14	2.5-3 mm (%) n = 10	P
ME	40.4 ± 9.4	27.2 ± 4.1	17.8 ± 6.4	< 0.01
MI	32.5 ± 8.9	18.8 ± 5.6	11.5 ± 3.7	< 0.01
IMH	9.9 ± 5.8	3.5 ± 2.4	0.6 ± 1.5	< 0.01

ME myocardial oedema; MI myocardial infarction; IMH intramyocardial haemorrhage

On T2* CMR, haemorrhage was evident in 25 rats (+ IMH group) on Day 2 and 16 rats (+ IMH group) on Day 7 (9 rats on Day 2 were killed for histological analysis). Based on LGE and T2*, a total of 167 imaging slices were identified as positive for MI (101 on Day 2 and 66 on Day 7) and 112 for IMH (66 on Day 2 and 46 on Day 7). No haemorrhage was observed in ten rats (–IMH group), and all rats on Day 2 were killed.

**Fig. 1:**
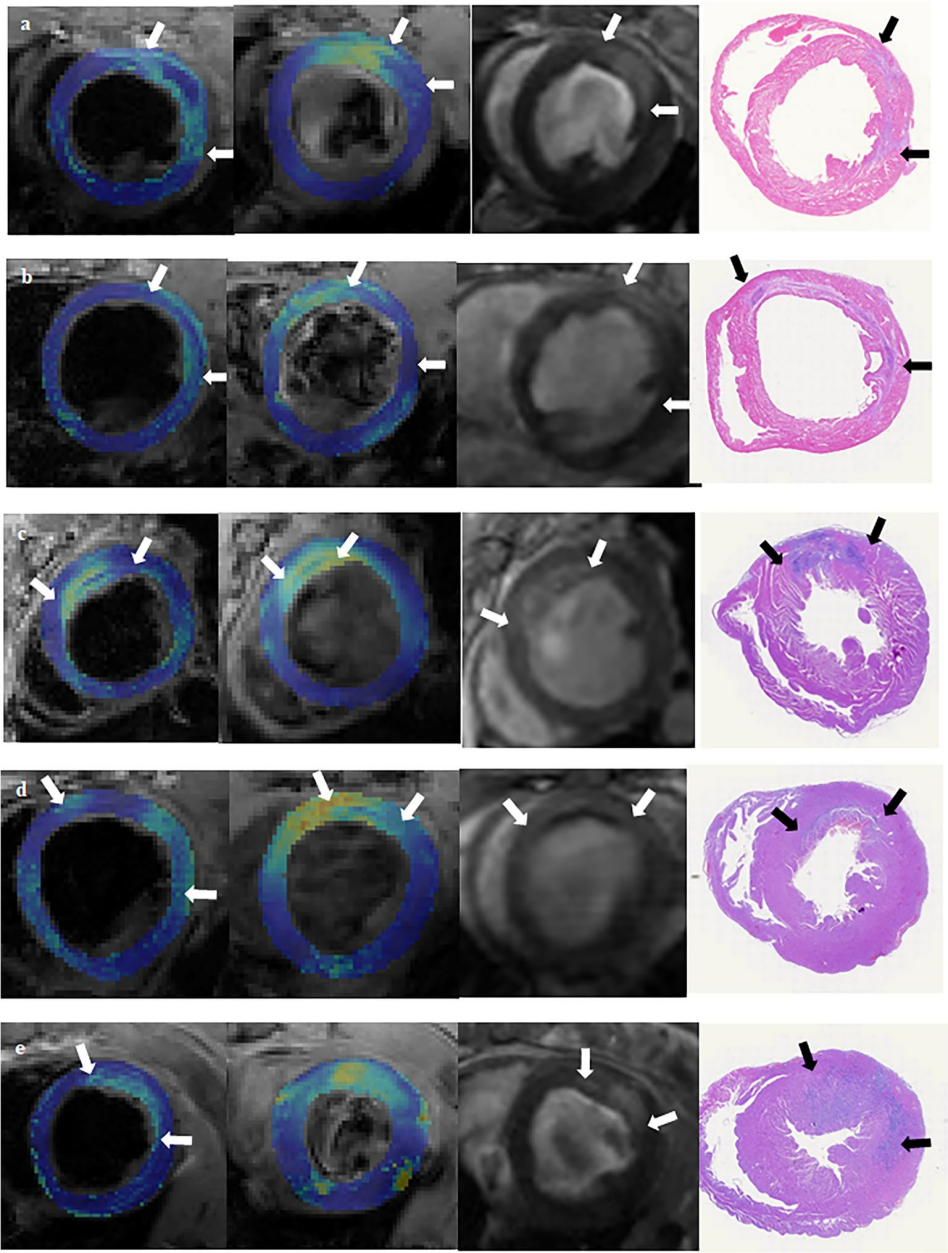
ME, IMH, MI and myocardial injury areas (arrows) showed by MRI (T2-mapping, T2* mapping, LGE) and H&E staining separately (from left to right). (a) Larger MI size in + IMH group at Day 2. (b) Larger MI size in + IMH group at Day 7. (c) Smaller MI size in + IMH group at Day 2. (d) Smaller MI size in + IMH group at Day 7. (e) Smaller MI size in -IMH group at Day 2. IMH, intramyocardial haemorrhage; MI, myocardial infarction; ME, myocardial oedema; LGE, late gadolinium enhancement; H&E, haematoxylin and eosin

Representative LGE images and corresponding noncontrast-enhanced T2*- and T2-maps from animals with and without haemorrhage on Days 2 and 7 of MI are shown in Fig. 1, respectively.

Mean T2* and T2 values of ME, MI, IMH, and remote regions, respective relative differences with respect to the remote myocardium (DT2 [%] and DT2* [%]), and absolute differences for T2 and T2* (DT2, DT2*), along with comparisons, are given in Table 2.

**Table 2:** T2*, T2, values of IMH, MI and ME territories and the associated absolute and percent relative differences of the variables with respect to remote myocardium in Day 2 and Day 7

2d	ME	MI	IMH	Remote
T2(ms)	35.6 ± 3.3	52.6 ± 9.2	33.8 ± 8.3	26 ± 2.9
∆T2(ms)	9.6 ± 2.7	26.8 ± 7.5	8.4 ± 7.4	
Relative ∆T2 (%)	37.4 ± 11.8	102.7 ± 26.6	31.8 ± 28.6	
T2*(ms)	17.9 ± 6.6	26.3 ± 10.3	16.3 ± 5.9	12.3 ± 4
∆T2*(ms)	5.6 ± 3.4	14 ± 7.6	4.2 ± 3.2	
Relative ∆T2*(%)	46.4 ± 22.5	119.5 ± 74.4	35.8 ± 27.4	
7d				
T2(ms)	32.7 ± 3.1(p > 0.05)	41.3 ± 4.5 (p < 0.01)	27.1 ± 6(p < 0.05)	24 ± 2.1(p > 0.05)
∆T2(ms)	8.7 ± 1.8(p > 0.05)	17.3 ± 3.6 (p < 0.01)	3.1 ± 5.5(p < 0.05)	
Relative ∆T2 (%)	36.3 ± 7.7(p > 0.05)	72.3 ± 15.5 (p < 0.01)	12.7 ± 23.6(p < 0.05)	
T2*(ms)	15.5 ± 2.8(p > 0.05)	21 ± 4.1( p < 0.05)	13.9 ± 2.7(p > 0.05)	10.6 ± 1.8(p > 0.05)
∆T2*(ms)	4.9 ± 2.2(p > 0.05)	10.4 ± 3.4(p < 0.05)	3.2 ± 2.9(p > 0.05)	
Relative ∆T2*(%)	47.4 ± 22.8(p > 0.05)	99.4 ± 33.9(p > 0.05)	33.2 ± 30.5(p > 0.05)	
-IMH group				
T2(ms)	39 ± 7.3 (p > 0.05)	49.7 ± 8.7 (p > 0.05)	-	27.1 ± 3.6(p > 0.05)
∆T2(ms)	11.9 ± 5 (p > 0.05)	22.6 ± 6.4 (p > 0.05)	-	
Relative ∆T2 (%)	43.8 ± 15.5 (p > 0.05)	83.5 ± 21.8 (p > 0.05)	-	
T2*(ms)	15.7 ± 3.6 (p > 0.05)	21.9 ± 4.8 (p > 0.05)	-	10.8 ± 1.3(p > 0.05)
∆T2*(ms)	4.9 ± 2.5 (p > 0.05)	11.1 ± 4.1 (p > 0.05)	-	
Relative ∆T2*(%)	44.5 ± 18.3 (p > 0.05)	102.4 ± 35 (p > 0.05)	-	

ME myocardial oedema; MI myocardial infarction; IMH intramyocardial haemorrhage

∆T2*, ∆T2 and relative ∆T2*, ∆T2 are as defined in text

Note. 7d p value denotes the changes from 2 to 7d. -IMH group p value denotes the differences between two groups

## Relationship in sizes of IMH, MI, and ME

**Fig. 2:**
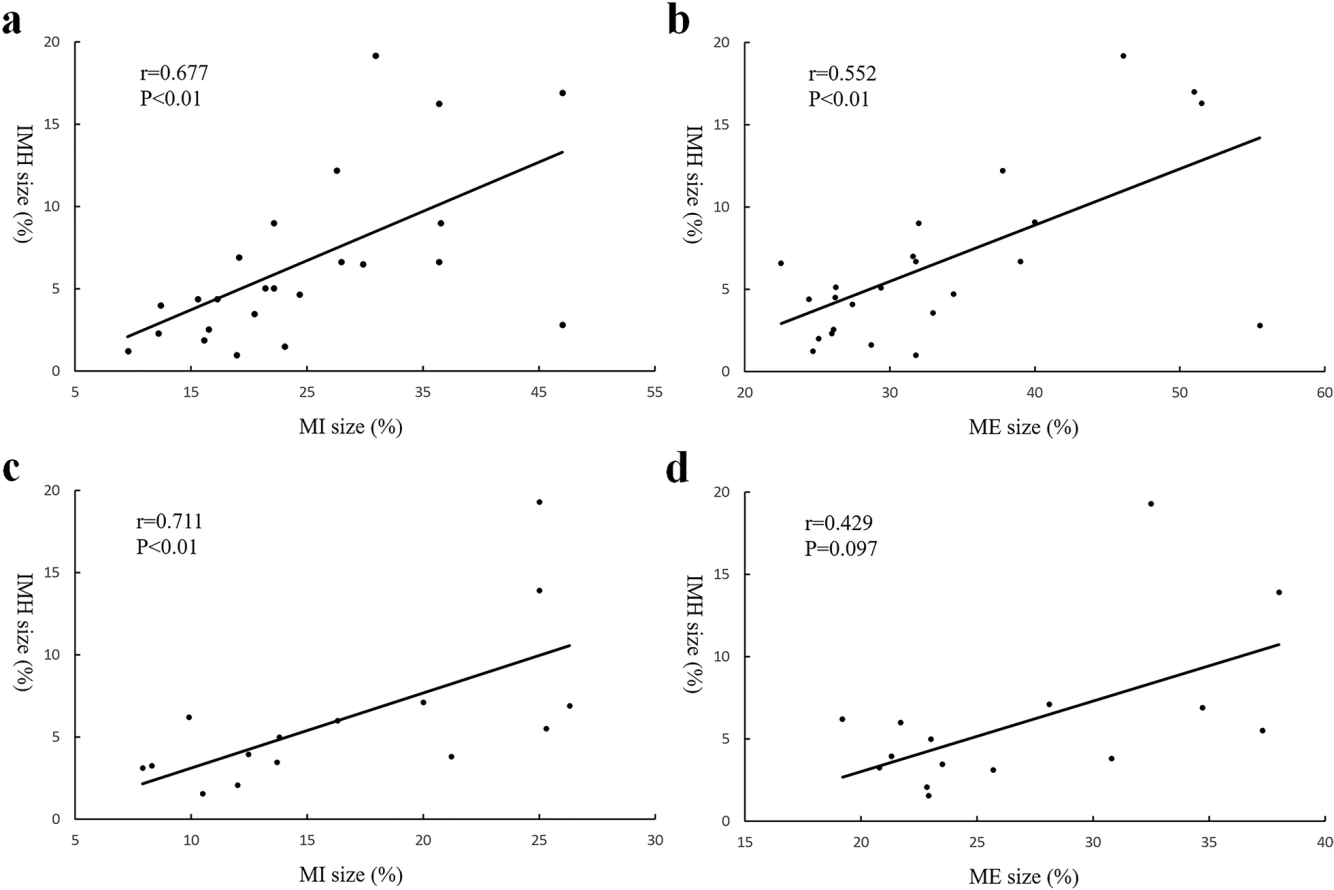
Relationship of IMH sizes and MI sizes or ME sizes in rats at Day 2 and Day 7. (a) Correlation between IMH sizes and MI sizes at Day 2. (b) Correlation between IMH sizes and ME sizes at Day 2. (c) Correlation between IMH sizes and MI sizes at Day 7. (d) Correlation between IMH sizes and ME sizes at Day 7. IMH, intramyocardial haemorrhage; MI, myocardial infarction; ME, myocardial oedema

On the 2^nd^ day, MI size ranged from 9.5% to 46.9%, IMH size ranged from 1.0% to 19.2%, and myocardial oedema size ranged from 22.5% to 55.5%. There was a significant positive correlation between IMH size and MI size (r = 0.677, P < 0.01) or ME size (r = 0.552, P < 0.01) (Fig. 2). The MI size and ME size of the + IMH group were significantly larger than those of the –IMH group (Table 3).

**Table 3:** The size parameters of the + IMH group and -IMH group

	2d (M(P_25_,P_75_))	7d (M(P_25_,P_75_))	P	-IMH group	P(vs + IMH2d)
ME	31.6(26.2,38.4)	23.7 (22,32.1)	< 0.01	17.3 ± 6.1	< 0.01
MI	22 (16.7,30.4)	13.8 (10.1,20.9)	< 0.01	12 ± 4.4	< 0.01
IMH	5.1 (2.7,9.1)	4.5 (3.1,6.7)	0.155	–	–

ME: myocardial oedema; MI: myocardial infarction; IMH: intramyocardial haemorrhage

On the 7th day, MI size ranged from 7.9% to 26.3%, IMH size ranged from 1.6% to 19.3%, and ME size ranged from 19.2% to 38%. There was a significant positive correlation between IMH size and MI size (r = 0.711, P < 0.01); however, there was no significant correlation between IMH size and ME size (r = 0.429, P = 0.097) (Fig. 2). Notably, from the 2nd to the 7th day, MI size and ME size decreased, while IMH size remained the same (Table 3).

## T2 and T2* values of IMH, MI, and ME

At Day 2, the T2 value of IMH was smaller than that of MI and larger than that of the remote area; however, there was no difference between IMH and ME, while on the 7th day, the T2 value of IMH was smaller than that of ME. From the 2nd to the 7th day, the T2 values of IMH and MI decreased, but there were no differences in the ME and remote regions.

The T2* value of IMH was smaller than that of MI and larger than that of the remote region; no difference was found between IMH and ME on the 2nd and 7th days. From the 2nd to 7th day, the T2* value of MI decreased, while there were no changes in ME, IMH, or remote region. The other parameters of the T2 and T2* values and the changes from the 2nd to the 7th day are shown in Table 2 and Table 4.

**Table 4:** P values of the differences of T2 and T2* parameters between IMH and other ROIs of + IMH group

	2d						7d					
ROIs	T2(ms)	∆T2(ms)	Relative∆ T2(%)	T2*(ms)	∆T2*(ms)	Relative∆ T2*(%)	T2(ms)	∆T2(ms)	Relative∆ T2(%)	T2*(ms)	∆T2*(ms)	Relative∆ T2*(%)
MI	0.01	0.01	0.01	0.01	0.01	0.01	0.01	0.01	0.01	0.01	0.01	0.01
ME	> 0.05	> 0.05	> 0.05	> 0.05	> 0.05	> 0.05	0.01	0.01	0.01	> 0.05	> 0.05	> 0.05
remote	0.01	-	-	0.05	-	-	0.05	–	-	0.01	-	-

ME: myocardial oedema; MI: myocardial infarction; IMH: intramyocardial haemorrhage

## Histopathology

Representative histopathologic images from rats with haemorrhage killed on the 2nd and 7th days and nonhaemorrhagic infarctions on the 2nd day of MI are shown in Fig. 1. In the + IMH group, H&E staining easily showed evidence of myocardial injury (myocyte necrosis), the distribution of inflammatory cells and haemorrhage. The –IMH group did not show evidence of haemorrhage on H&E staining.

## Discussion

This is one of a few studies using CMR to detect the relationship between the extent of MI and haemorrhage in small animal models. We observed the following: 1) a small IMH size in the small infarction area; 2) a positive linear relationship between the size of infarction and haemorrhage; 3) the MI area was the highest T2 value region, which decreased from 2 to 7 d; and 4) the T2 value of IMH decreased from Day 2 to 7, while the size and T2* remained the same.

The infarction size was determined for several reasons. One reason was the position of occlusion of the coronary artery. One minor discrepancy in the exact location along the LAD where the occlusion was introduced could lead to a variation in myocardial infarction size [22]. In our study, we used three positions of ligating to mimic clinical MI in patients, while other studies always used approximately 2 mm from its origin. We found that in the lowest position of ligating, while the infarction size was the smallest, IMH only occurred in two rats with 2% and 4.5% of the entire LV in this group, while 100% (11/11) and 85.7% (12/14) IMH occurred in the highest and middle position ligating groups, respectively.

A patient study revealed a relationship between IMH and MI (r = 0.45 or 0.67); however, the size of MI was 28.5% LV in this study [19]. A recent patient study also reported a relationship between IMH and necrosis (r = 0.58), and the size of MI in this study was 24 ± 10% [20]. In a canine study, there was a moderate correlation between the extent of necrosis and the extent of haemorrhage (r = 0.56; p < 0.05) [2], and the size of MI was 19.3 ± 4% LV. A recent canine study found a good correlation between the size of IMH and MI (r = 0.73 or 0.86; p < 0.05) with a relatively small sample size (n = 13 or 17) [21].

Several patient studies found infarction size with IMH was larger than those of the non-IMH [2, 7, 9–22] (Table 5). In a rat study, the sizes of MI with IMH were bigger than those without IMH [22]. However, another canine study reported infarction size (23% LV) with IMH was not different than non-IMH in reperfused acute MI (19.7% LV) [15]. A possible explanation for this might be the small sample size (n = 6 in IMH group). Our study confirmed that the sizes of the myocardial infarction in the + IMH group were larger than those in the -IMH group, consistent with most previous studies.

**Table 5:** The previous infarction sizes of comparisons between IMH and non-IMH patients and animals

Reporters	Infarction size of IMH group (%LV)	Year
O'Regan DP et al.	23.8 ± 8.2	2010
Kumar A et al.	38.8 ± 2.8	2011
Mather AN et al.	36.2	2011
Kandler D et al.	33 ± 20 ml (%LV not available)	2014
Ding S et al.	32.6 ± 11.6	2015
Durighel G et al.	25 (19–28)	2016
Carrick D et al.	29	2016
Carrick D et al.	22–30	2016
Bulluck H et al.	33.4 ± 11.3	2017
Amier RP, et al.	16.9–45.4	2017
Robbers LFHJ et al.	25 ± 11	2018
Ma M et al.	36.5 ± 14.8	2018
Nair AR	34 ± 8	2020
Ferré-Vallverdú M et al.	24 ± 10	2021
Liu T et al.	36.6 ± 13.2	2022

IMH: intramyocardial haemorrhage

In our study, we designed different extents of MI from 9.5% to 46.9%. This infarction extent was larger than in former studies [2, 7, 9–22]; the range of these studies was not comprehensive enough to reveal the relationship between IMH and MI. Some areas in our study were smaller than those in other studies, suggesting that IMH can be found in small MI sizes. This positive correlation suggests that these components of tissue injury are not independent of each other and that there could perhaps be a causal relationship. Haemorrhage occurred mainly in infarcts with the largest infarct size, suggesting that the amount and severity of tissue injury may be a predisposing factor for the development of haemorrhage. Multiple mechanisms contribute to these manifestations; coronary artery occlusion followed by reperfusion causes severe capillary damage, and erythrocytes leak out of endothelial gaps that result in IMH, which is not only a by stander phenomenon; extravasation of erythrocytes, leucocytes and finally iron deposition further increase myocardial damage via a sustained inflammatory reaction [28–30].

Myocardial haemorrhage represents a hypointense infarct core with a T2* value of < 20 ms [7] or mean-2SD [31]; in this study, the T2* value of IMH was 3.2–4.2 ms more than the remote area, and there was no difference with the ME region. This is not consistent with the former study, while the mean T2* of the haemorrhagic area was lower than that of the remote myocardium, which might be due to the different sequences and reperfusion times. Although T2* mapping is the most sensitive method for imaging haemorrhage, the spatial resolution is relatively low, and it is sensitive to motion and blood flow [31], so we ensured that the + IMH group included IMH that was visible on both T2* imaging and pathological findings.

A recent ^19^F-CMR study found inflammation of MI more than IMH, helping us explain why the T2 value of MI was larger than that of IMH in this study [32]. The persistently high T2 value of MI reflected inflammation around IMH found in this study confirms previous results and indicates that haemorrhage prolongs [33], if not prevents, recovery after 7 days. Our findings are consistent with a former study showing that the no-reflow persists for at least 1 month after reopening of the epicardial coronary artery [34]. The MI size decreased from Day 2 to Day 7, except for the real myocardial infarction area; another possible explanation for this is that the infarct zone contains a heterogeneous pathology area in early post-MI.

## Limitations of the study

First, there were no quantitative histological data of IMH size; haematoxylin–eosin staining could qualitatively detect IMH to some extent, but we lacked an accurate method for the entire IMH area. Second, TTC staining was not performed to confirm infarction size because the entire HE staining was more necessary to confirm the IMH area, and we confirmed that there was no significant difference between the infarction size measured by our LGE sequence and TTC staining in the former study [26, 27]. Last, this study reported IMH only, not MVO because FISP-cine has a relatively long acquisition time in the present study, which may underestimate the extent of MVO, and a total of 72 imaging slices were identified as positive for MVO (47 on Day 2 and 25 on Day 7) based on LGE in this study.

## Conclusions

In conclusion, our data suggest that there was a positive relationship between IMH and MI, and the small infarction size could also lead to small IMH, which could not be easily dissolved in the early phase of reperfusion. Myocardial infarction volume could be an independent predictor of intramyocardial haemorrhage volume for percutaneous coronary intervention of myocardial ischaemic patients.

## Data Availability

The datasets used and/or analysed during the current study are available from the corresponding author on reasonable request.

## Abbreviations

MI: Myocardial infarction
IMH: Intramyocardial haemorrhage
ME: Myocardial oedema
CMR: Cardiovascular magnetic resonance
MVO: Microvascular obstruction
LAD: Left anterior descending
ECG: Electrocardiogram
MSME: Multislice multiecho
MGE: Multigradient-echo
FISP: Fast imaging with steady-state precession
TR: Time of repetition
TE: Time of echo
FOV: Field of view
FA: Flip angle
